# Lipoprotein (a) as a residual risk factor for atherosclerotic renal artery stenosis in hypertensive patients: a hospital-based cross-sectional study

**DOI:** 10.1186/s12944-020-01272-0

**Published:** 2020-07-23

**Authors:** Xiangming Hu, Xing Yang, Xida Li, Demou Luo, Yingling Zhou, Haojian Dong

**Affiliations:** 1 Department of Cardiology, Guangdong Provincial People’s Hospital, Guangdong Academy of Medical Sciences, #96 Dongchuan Road, Guangzhou, 510080 Guangdong China; 2 The Second School of Clinical Medicine, Southern Medical University, Guangzhou, 510515 Guangdong China; 3 Department of Cardiology, Guangdong Provincial People’s Hospital Zhuhai Hospital (Zhuhai Golden Bay Center Hospital), Zhuhai, 519040 Guangdong China; 4 Department of Cardiology, Vascular Center, Guangdong Cardiovascular Institute, Guangdong Provincial Key Laboratory of Coronary Heart Disease Prevention, Guangdong Provincial People’s Hospital, Guangdong Academy of Medical Sciences, #96 Dongchuan Road, Guangzhou, 510080 Guangdong China

**Keywords:** Lipoprotein (a), Atherosclerotic renal artery stenosis, LDL-cholesterol, Residual risk

## Abstract

**Background:**

Low-density lipoprotein cholesterol (LDL-c) has been proven to be a risk factor for atherosclerotic cardiovascular disease (CVD), while lipoprotein (a) (Lp(a)) is a residual risk factor for CVD, even though LDL-c is well controlled by statin use. Importantly, the role of Lp(a) in atherosclerotic renal artery stenosis (ARAS) is still unknown.

**Methods:**

For this hospital-based cross-sectional study, patients who simultaneously underwent coronary and renal angiography were examined. ARAS was defined as a 50% reduction in the cross-sectional (two-dimensional plane) area of the renal artery. Data were collected and compared between ARAS and non-ARAS groups, including clinical history and metabolite profiles. Univariate analysis, three tertile LDL-c-based stratified analysis, and multivariate-adjusted logistic analysis were conducted, revealing a correlation between Lp(a) and ARAS.

**Results:**

A total of 170 hypertensive patients were included in this study, 85 with ARAS and 85 with non-RAS. Baseline information indicated comparability between the two groups. In the univariate and multivariate analysis, common risk factors for atherosclerosis were not significantly different. Stratified analysis of LDL-c revealed a significant increase in the incidence of ARAS in patients who had high Lp(a) concentrations at low LDL-c levels (odds ratio (OR): 4.77, 95% confidence interval (CI): 1.04–21.79, *P* = 0.044). Further logistic analysis with adjusted covariates also confirmed the result, indicating that high Lp(a) levels were independently associated with ARAS (adjusted OR (aOR): 6.14, 95%CI: 1.03–36.47, *P* = 0.046). This relationship increased with increasing Lp(a) concentration based on a curve fitting graph. These results were not present in the low and intermediate LDL-c-level groups.

**Conclusion:**

In hypertensive patients who present low LDL-c, high Lp(a) was significantly associated with atherosclerotic renal artery stenosis and thus is a residual risk factor.

## Background

Cardiovascular disease (CVD) is a leading cause of deaths in China and a large proportion of CVD cases are caused by arteriosclerotic cardiovascular disease (ASCVD). The number of deaths arising from ASCVD has rapidly and substantially increased, and it was responsible for > 2.4 million deaths in 2016 and accounting for 25% of total deaths [1]. Mendelian randomization studies and RCTs have consistently demonstrated that low-density lipoprotein cholesterol (LDL-c) is causally associated with the risk of ASCVD [2–7]. The American Heart Association/American College of Cardiology and European Society of Cardiology guidelines provide recommended LDL-c levels based on CVD risk stratification. In a recent ESC article, lipoprotein (a) [Lp(a)] was highlighted as a CVD risk estimator [8]. Studies from the past few decades have revealed that populations with well-regulated LDL-c levels still had a considerably high residual cardiovascular risk, and that Lp(a) is responsible for this phenomena [9–14].

Atherosclerotic renal artery stenosis (ARAS), which represents a considerable proportion of ASCVD cases, is generally recognized to cause renal damage and accounts for 5–15% of patients who develop end-stage renal disease [15–17]. The incidence of symptomless ARAS has been reported to be high in patients undergoing angiography for extrarenal atherosclerotic vascular disease, especially in hypertensive patients [18], reflecting the prevalence of ARAS in systemic atherosclerosis, and it is usually overlooked [19, 20]. Hypertension can accelerate the progress of ARAS by facilitating lipid deposition, in addition to other traditional CVD risk factors, such as age [21, 22], diabetes [23] and smoking [24], which are also related to ARAS. Therefore, the question: does Lp(a) act as a “residual risk” factor for ARAS in hypertensive patients? is worthy to explore. Recent studies have suggested a relationship between Lp(a) and ARAS [25, 26], but further evidence is required to clarify this relationship, which is the aim of this study.

## Methods

### Study population and data collection

This study was designed as a cross-sectional analysis. From October 2013 to September 2014, patients with hypertension who had simultaneously undergone both coronary and renal angiography with hypertension were consecutively selected from a single catheter center in China. Initially, patients underwent coronary angiography because of suspected severe coronary artery disease (CAD), and renal angiography was also performed if the patient satisfied any of the following conditions: patients who developed hypertension before age 30; patients who developed severe hypertension after age 55; patients with rapid, refractory, malignant, or suddenly aggravated hypertension; patients with deteriorated renal function (as marked by a > 30% increase in serum creatinine) after treatment with angiotensin-converting enzyme inhibitors or angiotensin receptor blockers; patients with unexplained renal atrophy or > 1.5-cm difference in length of kidney; patients with unexplained sudden exacerbated and/or refractory pulmonary edema; patients with coronary multivessel disease, cerebrovascular disease, or peripheral atherosclerotic disease; patients with unexplained exacerbation of renal failure (including patients undergoing dialysis or kidney transplantation); or patients with unexplained congestive heart failure. Exclusion criteria included a history of cancer, coagulation disorder, or renal stenting.

All experimental data were collected from the case database of the medical center and recorded by two authors (Yang and Li).

### Definitions and laboratory examination

In all patients, hypertension was diagnosed according to the European Society of Cardiology guidelines as SBP ≥ 140 and/or DBP ≥ 90 mmHg, which is equivalent to a 24-h ambulatory blood pressure monitoring average of ≥130/80 mmHg, or a home blood pressure monitoring average of ≥135/85 mmHg for two measurements at least 3 days [27]. Diabetes status was diagnosed based on presence of diabetes. If the patient had a negative history of diabetes, the repeatedly fasting blood glucose ≥7.0 mmol/L (126 mg/dL) or haemoglobin A1c ≥ 6.5% or oral glucose tolerance test positive test (2 h plasma glucose ≥11.1 mmol/L (200 mg/dL)) were adopted to define diabetes status according to the European Society of Cardiology guidelines [28]. Blood cell test was detected using a Sysmex-XE5000 through impedance technology. HDL-cholesterol, LDL-cholesterol, total-cholesterol, Lp(a), albumin, uric acid, creatinine, and cystatin C were detected using a Backman AU5800 spectrophotometer via colorimetry or immunoturbidimetry. Aldosterone, renin, and angiotensin II were detected using a PETECK96-I through a chemiluminescence immunoassay. The evaluated glomerular filtration rate (eGFR) (mL·min^− 1^·1.73 m^− 2^) was calculated using the Cockroft–Gault formula.

Coronary and renal angiography was performed by the Judkins technique. CAG and renal angiography were performed simultaneously with radial approach, and the femoral artery was used in a minority of patients as clinically necessary. Catheter 5-Fr or 6-Fr Judkins left and right diagnostic catheters (Cordis, Bridgewater, NJ, USA) were used for left and right coronary angiography, respectively. Renal angiography was performed using a 5-Fr Judkins right or 5-Fr Multi-Purpose diagnostic catheter engaged in or directed to the renal artery ostium, with contrast medium flowing back from the renal artery. Both renal arteries were visualized in anterior-posterior projections. All angiograms were independently reviewed by an experienced angiographer. Lesion severity in the coronary tree and the renal vasculature was assessed by visual estimation. ARAS was defined as a 50% reduction in the area of cross-sectional or two-dimensional plane of the renal artery, as presented by renal arterial lumen loss (RALL) ≥ 50%. As suggested by American College of Cardiology and Chinese Cardiovascular Disease Association in 2016 [29, 30], a ≥ 70% luminal diameter narrowing of an epicardial stenosis or ≥ 50% luminal diameter narrowing of the left main artery made by visual assessment were regarded as severe CAD to identify those with high-risk atherosclerotic factors. Peripheral arterial disease (PAD) was defined by one or more of the following conditions: intermittent claudication symptoms; previous surgery for lower limb arterial; angiography showing the presence of significant stenosis in the lower limbs/subclavian/carotid/vertebral artery and abdominal aortic aneurysm.

### Statistical analysis

Statistical analysis was performed in three steps. First, the baseline characteristics of the participants were measured according to following principles after they were divided into two groups (ARAS and non-ARAS): (1) continuous variables were expressed as the means ± standard deviations (for normal distribution) or medians/quartiles (for skewed distribution), and categorical variables were shown as the frequencies with percentages; (2) T-test for normal distribution data, Mann-Whitney U test for skewed distribution data, and chi-square test/Fisher’s exact test for categorical variables were used to determine significant differences between the groups. Next, univariate and multivariate analysis were conducted to find potential risk factors. Then, given that until now, there is no clear standard for stratification related to Lp(a), an LDL-c-based stratified analysis was conducted to assess the relation between Lp(a) and ARAS. Finally, age, gender, BMI, current smoking status and DM that regarded as common atherosclerosis risk factors were pooled for multivariate adjustment by logistic analysis and used to assemble generalized additive models to identify non-linear relationships where Lp(a) was a continuous variable. If an incremental effect model was present, it was trimmed into three tertiles to determine the threshold point for risk assessment. Comparisons where *P* < 0.05 (two-sided) were considered to be statistically significant. All of the analyses were performed with Stata 15.0, R (version 3.4.3) and EmpowerStats (http://www.empowerstats.com, X&Y Solutions, Inc., Boston, MA).

## Result

### Baseline information

A total of 170 hypertensive patients were analyzed in this study. Based on the RALL range, these patients were divided into two groups: ARAS (RALL ≥50%) and non-ARAS (RALL < 50%). All baseline characteristics are included in Table 1. The median age of the participants was 69 years and male accounted for 64.71% of the study population. Of these, 22 patients had bilateral renal artery stenosis, 63 patients had unilateral renal artery stenosis, and 85 patients did not have renal artery stenosis. SBP, CAD, PAD and calcium channel blockers used were found to be significantly different between two groups. Table 2 details the metabolites levels for the patients, in which creatinine, eGFR, and aldosterone were significantly different between the patient groups.

**Table 1:** Baseline characteristics of ARAS and non-ARAS patients

	TOTAL *N* = 170	ARAS *N* = 85	Non-ARAS *N* = 85	*P* value
Age (year)	69.00 (62.00 – 75.00)	72.00 (64.00 – 76.00)	68.00 (61.00 – 74.00)	0.062
Male	110 (64.71)	57 (67.06)	53 (62.35)	0.521
Body Mass Index (kg/m2)	23.93 (22.15 – 25.82)	23.73 (21.64 – 25.78)	24.03 (22.59 – 26.12)	0.214
Current smoking	51 (30.00)	30 (35.29)	21 (24.71)	0.132
Systolic blood pressure (mmHg)	147.08 ± 24.01	151.72 ± 26.66	142.44 ± 20.14	0.011
Diastolic blood pressure (mmHg)	77.64 ± 11.57	77.79 ± 11.58	77.48 ± 11.63	0.864
Diabetes mellitus	68 (40.00)	33 (38.82)	35 (41.18)	0.754
Coronary artery disease	139 (81.76)	63 (74.12)	76 (89.41)	0.010
Peripheral arterial disease	43 (25.29)	28 (32.94)	15 (17.65)	0.022
Antihypertensive	166 (97.65)	83 (97.65)	83 (97.65)	1.000*
ACEIs/ARBs	134 (78.82)	62 (72.94)	72 (84.71)	0.060
β-receptor blockers	121 (71.18)	59 (69.41)	62 (72.94)	0.611
Calcium channel blockers	84 (49.41)	51 (60.00)	33 (38.82)	0.006
Diuretics	47 (27.65)	23 (27.06)	24 (28.24)	0.864
α-receptor blockers	11 (6.47)	9 (10.59)	2 (2.35)	0.057*
Statin	10 (5.88)	4 (4.71)	6 (7.06)	0.746*

Abbreviations: *ACEIs* Angiotensin converting enzyme inhibitors, *ARBs* Angiotensin receptor blockers

*Fisher’s exact test

**Table 2:** Metabolites in ARAS and non-ARAS patients

	TOTAL *N* = 170	ARAS *N* = 85	Non-ARAS *N* = 85	*P* value
Total-cholesterol (mmol/L)	4.50 (3.77 – 5.41)	4.50 (3.80 – 5.31)	4.52 (3.68 – 5.58)	0.437
Triglyceride (mmol/L)	1.42 (1.02 – 2.04)	1.38 (1.05 – 2.02)	1.46 (0.99 – 2.12)	0.410
HDL-cholesterol (mmol/L)	0.97 (0.83 – 1.15)	0.98 (0.83 – 1.15)	0.96 (0.83 – 1.15)	0.821
LDL-cholesterol (mmol/L)	2.66 (2.11 – 3.34)	2.66 (1.97 – 3.20)	2.62 (2.20 – 3.49)	0.272
Lipoprotein (a) (mg/L)	171.73 (79.50 – 376.75)	171.00 (74.73 – 535.75)	172.46 (95.00 – 322.00)	0.173
Hemoglobin (g/L)	125.50 ± 18.89	124.88 ± 20.08	126.12 ± 17.72	0.668
Platelet (*10^9/L)	210.65 (178.00 – 259.50)	209.40 (174.00 – 244.00)	211.00 (180.00 – 263.00)	0.206
Albumin (g/L)	35.79 (32.70 – 38.20)	35.70 (33.40 – 38.40)	35.90 (32.52 – 37.88)	0.378
Uric Acid (μmol/L)	408.95 (331.50 – 482.25)	408.90 (324.00 – 489.00)	409.00 (339.00 – 477.50)	0.921
Creatinine (μmol/L)	97.50 (77.67 – 136.75)	105.48 (87.40 – 148.30)	89.00 (68.43 – 120.00)	0.008
Cystatin C (mg/L)	1.23 (0.99 – 1.56)	1.25 (1.03 – 1.77)	1.15 (0.91 – 1.54)	0.056
eGFR (ml/(min·1.73m^2^))	56.03 (38.82 – 72.04)	47.97 (34.91 – 64.94)	62.70 (44.53 – 77.98)	< 0.001
Aldosterone (nmol/L)	0.29 (0.20 – 0.50)	0.33 (0.22 – 0.53)	0.24 (0.19 – 0.42)	0.048
Renin (nmol/L)	0.62 (0.21 – 1.70)	0.71 (0.28 – 2.00)	0.46 (0.13 – 1.09)	0.194
Angiotensin-II (ng/L)	46.50 (35.00 – 82.55)	48.00 (36.00 – 83.00	45.00 (34.40 – 81.00)	0.372

### Univariate, multivariate and stratified analysis

We conducted both univariate and multivariate analysis of ARAS the results were shown in Table 3. In the univariate and multivariate analysis, age, gender, body mass index (BMI), current smoking status, Lp(a), LDL-c and diabetes mellitus (DM) were not associated with ARAS. Sensitivity analysis also found that previous antihypertensive and lipid-lowering therapy were not associated with ARAS (Additional file 1: Table S1). In order to explore the relationship between Lp(a) and ARAS in a low LDL-c population, a stratified analysis was performed using three tertiles. In a low LDL-c population (≤ 2.29 mmol/L), patients with high Lp(a) levels had significantly higher rates of ARAS than patients with low Lp(a) levels (Table 4, odds ratio (OR): 4.77; 95% confidence interval (CI): 1.04–21.79; *P* = 0.044).

**Table 3:** Univariate and multivariate analysis for ARAS

Variable	Univariate		Multivariate	
	OR (95%CI)	*P* value	OR (95%CI)	*P* value
Age	1.03 (1.00, 1.07)	0.064	1.03 (0.99, 1.06)	0.123
Male	1.23 (0.65, 2.31)	0.521	0.97 (0.47, 2.01)	0.937
Body Mass Index	0.94 (0.86, 1.03)	0.216	0.97 (0.88, 1.07)	0.511
Current smoking	1.66 (0.86, 3.23)	0.134	1.67 (0.79, 3.56)	0.182
Lp(a) tertile				
Low	1.0		1.0	
Intermediate	0.54 (0.26, 1.15)	0.109	0.55 (0.25, 1.20)	0.134
High	1.07 (0.51, 2.25)	0.851	1.14 (0.53, 2.48)	0.733
LDL-c tertile				
Low	1.0		1.0	
Intermediate	0.75 (0.36, 1.58)	0.455	0.78 (0.36, 1.68)	0.523
High	0.63 (0.30, 1.32)	0.226	0.70 (0.32, 1.54)	0.370
Diabetes mellitus	0.91 (0.49, 1.68)	0.754	0.76 (0.40, 1.46)	0.408

Illustration: “Low” (OR = 1) as the reference

**Table 4:** LDL-c-based stratified analyses for Lp(a) and ARAS by three tertiles

Variable	OR (95%CI)	P value
Lp(a) tertile in low LDL-c subgroup		
Low	1.0	
Intermediate	0.90 (0.26, 3.07)	0.867
High	4.77 (1.04, 21.79)	0.044
Lp(a) tertile in intermediate LDL-c subgroup		
Low	1.0	
Intermediate	0.17 (0.04, 0.81)	0.026
High	0.57 (0.17, 1.96)	0.374
Lp(a) tertile in high LDL-c subgroup		
Low	1.0	
Intermediate	0.77 (0.20, 2.92)	0.700
High	0.75 (0.19, 2.92)	0.678

Illustration: “Low” (OR = 1) as the reference

### Logistic analysis

**Fig. 1:**
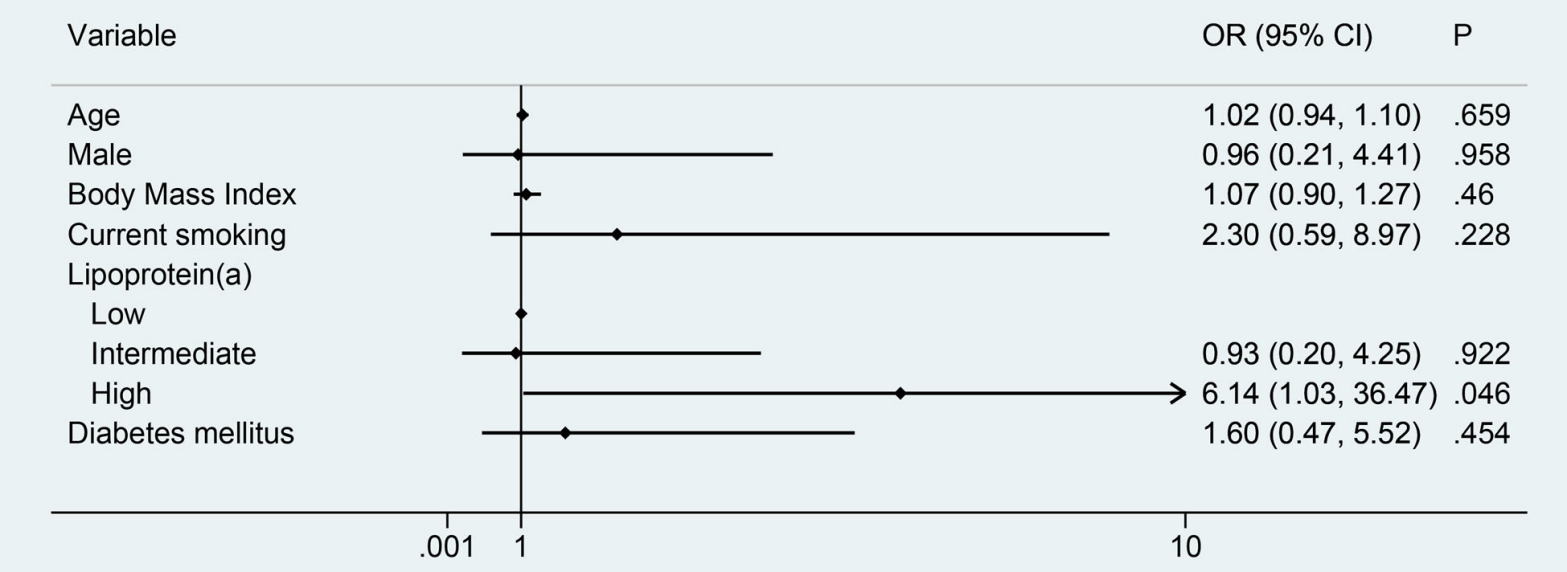
Forest plot for multivariate analysis with ARAS in a low LDL-c population by logistic regression

A logistic analysis was performed to identify additional risk factors besides Lp(a) among populations with low LDL-c levels. The incidence of ARAS dramatically increased in patients with high Lp(a) levels after adjusting for other influence (adjusted OR (aOR): 6.14, 95%CI: 1.03–36.47, *P* = 0.046), including age, gender, BMI, current smoking status and DM (Fig. 1). In the sensitivity analysis by including the previous antihypertensive and lipid-lowering therapy for adjusting, we found the association between Lp (a) and ARAS remained the same with aOR = 4.13 in low level of LDL-c patients.

### The relationship between Lp(a) and ARAS in a low LDL-c population

**Fig. 2:**
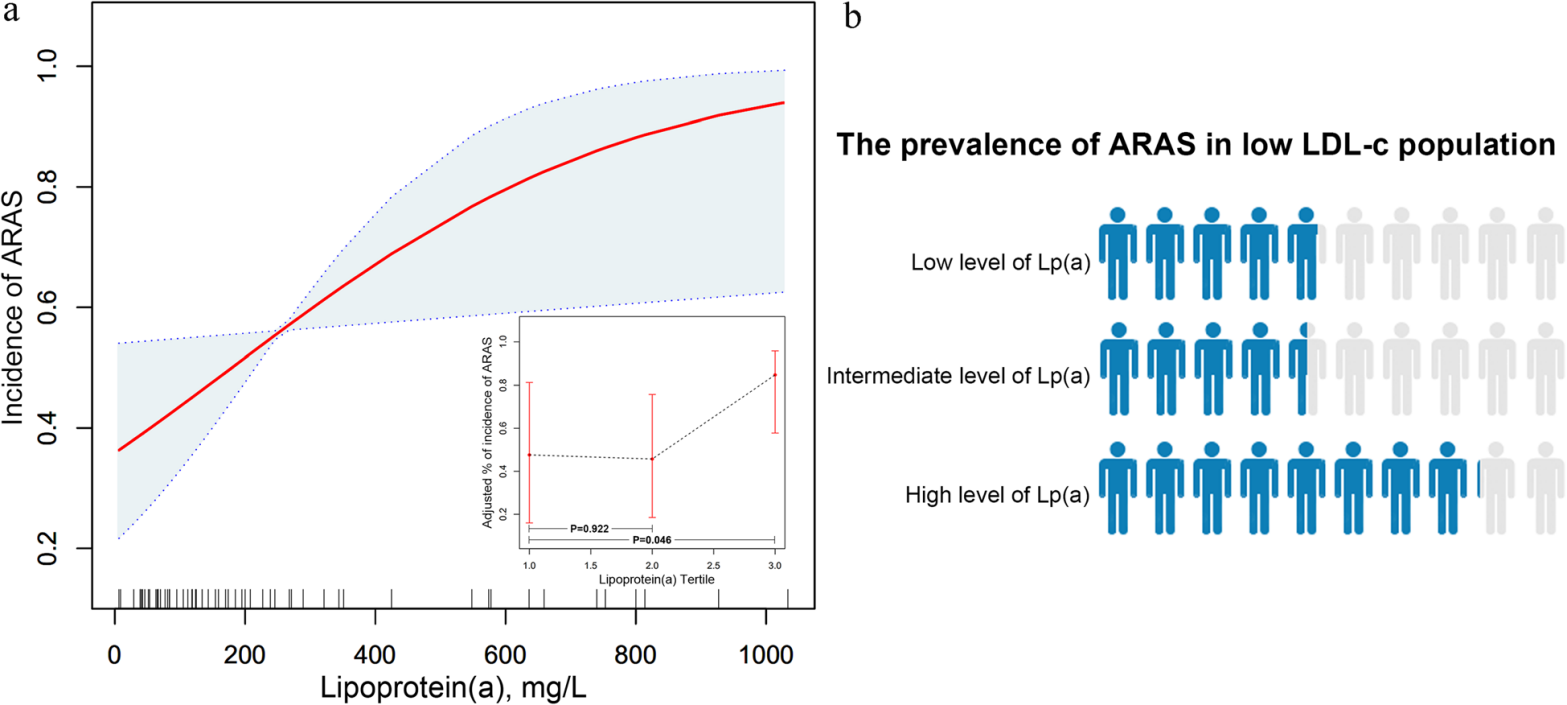
**a.** Non-linear relationship and tertile points between Lp(a) and ARAS adjusted covariates. Illustration: External image: the x-axis is Lp(a) concentration. The y-axis is the incidence of ARAS, with the shaded area representing a 95% confidence interval. (linear trend, *P* = 0.028). Internal image: the x-axis is Lp(a) concentration. The y-axis is the incidence of ARAS when dividing Lp(a) concentrations into three tertiles. The reference group (low Lp(a)) was set to 1.0. **b.** Population-based ARAS prevalence corresponding to different concentrations of Lp(a) levels in patients with low LDL-c levels. Illustration: Blue color indicates the prevalence of ARAS at different Lp(a) levels among a low LDL-c population.

Based on the logistic regression analysis, the incidence of ARAS was set as the endpoint, with the Lp(a) concentration acting as the main influence factor in plotting the fitting graph and adjusting for other covariates. The relationship between Lp(a) and ARAS was non-linear, with ARAS levels leveling off at a certain concentration of Lp(a) (Fig. 2a). As Lp(a) was a continuous variable, three points could be used to represent different thresholds of morbidity. Compared with the low Lp(a) concentration group, the high Lp(a) concentration group was significantly related to the incidence of ARAS (*P* = 0.046), while differences in comparison to the intermediate group were not significant (*P* = 0.922). The probability of a patient with low LDL-c levels suffering from ARAS was calculated for different levels of Lp(a) (Fig. 2b).

### The distribution of Lp(a) in population

**Fig. 3:**
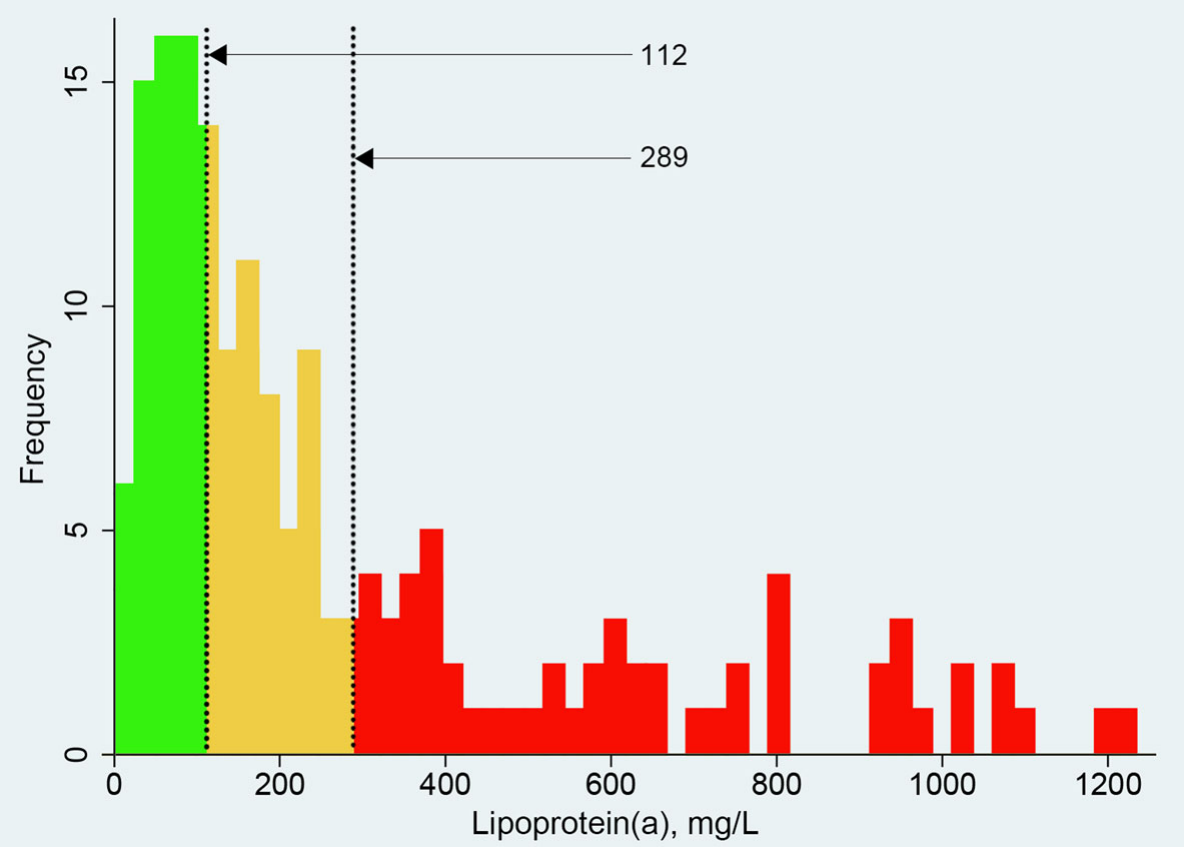
Distribution of Lp(a) concentrations in a population. *Illustration: Red bars (Lp(a) > 289 mg/L) represent* increased *possibility of suffering from ARAS at low LDL-c levels*

Setting Lp(a) concentration as the continuous variable, the concentration demarcation point was set using the Lp(a) tertile method on the study population in order to obtain risk stratification. The distribution of Lp(a) was positively skewed to the right, and ARAS risk was significantly increased in the upper tertile in low LDL-c patients (Fig. 3).

## Discussion

This cross-sectional study indicated that, in a hypertensive Chinese population with low levels of LDL-c, Lp(a) was identified as a significant residual risk factor for ARAS.

The result was performed in five parts. Firstly, in univariate analysis and multivariate analysis, age, gender, BMI, current smoking status, Lp(a), LDL-c and DM were not associated with ARAS. Secondly, we analyzed different concentrations of Lp(a) in a low LDL-c population, revealing that high-levels of Lp(a) were associated with a high incidence rate of ARAS, further supporting the hypothesis that ARAS and Lp(a) levels are related. Next, logistic analysis that adjusted for other covariates in this low LDL-c population to further confirmed the hypothesis. After controlling for age, gender, BMI, current smoking and DM, we found that there was a significant effect of Lp(a) on ARAS in a low LDL-c population. Subsequently, in order to more intuitively demonstrate this relationship with ARAS, a concentration-prevalence fitting curve was plotted, revealing that incremental increases in Lp(a) concentration of Lp(a) initially caused increased ARAS levels, before leveling off at a certain rate. At the same time, Lp(a) concentration was divided into three tertiles in order to generate a line chart to estimate risk proportions. Finally, the distribution of Lp(a) concentrations and the tertile Lp(a) point in hypertensive patients were analyzed to distinguish different population based on ARAS risk. To our knowledge, we are the first to demonstrate an independent association between Lp(a) concentration and ARAS in the hypertensive low LDL-c population.

Pathophysiologically, the mechanisms by which Lp(a) increases CVD risk are driven by proatherogenic and prothrombotic states, including endothelial disorder, smooth muscle proliferation, foam cell formation, and local coagulation disturbances [13]. Molecularly, Lp(a) is similar to LDL-c, as it is a particle covalently bound by apoB and apo (a), which carries pathogenic LDL-c and leads to atherosclerosis [31]. However, Lp(a) is more atherogenic than LDL-c due to the presence of apo (a), which can induce inflammation that is mediated by oxidized phospholipids and antifibrinolytic effects that result from inhibiting plasminogen activation [31–34]. Lp(a) shares similarities to LDL-c, which may account for the associated risk of Lp(a) leading to atherosclerosis initiation and progression in a low LDL-c environment. In this study, Lp(a) levels were significantly associated with ARAS at low LDL-c levels. One explanation for this effect is that the impact of Lp(a) is reduced at high LDL-c concentrations. Although Lp(a) has a stronger pathogenicity, LDL-c is still a significant factor in atherosclerosis progression. Together, this underscores the importance of Lp(a) in the context of low LDL-c levels and promotes further study of the related residual risks.

Clinical trials and systematic reviews over the past several decades have revealed a strong relationship between Lp(a) concentration and CVD [35–39]. For example, the JUPITER trial of low LDL-c participants demonstrated that baseline Lp(a) concentrations were associated with increased CVD risk [14]. Similar results were obtained from AIM-HIGH and LIPID trials in which participants underwent LDL-c lowering therapy [40, 41]. These data suggest that high Lp(a) levels act as a latent pathogenic factor during the development and treatment of CVD wherein common risks are treated. This study examining the relationship between ARAS and Lp(a) supports these observations, indicating that Lp(a) is a determinant for residual risk in hypertensive patients with low LDL-c levels. In the general population, *LPA* is the major gene controlling the Lp(a) feature and explains 70–90% of the variance in Lp(a) levels [42]. In this study, most patients had undergone primary angiographic without statin treatment, so their baseline Lp(a) levels were mostly controlled by genetics, suggesting that the study’s results are applicable to those with naturally high Lp(a) levels. This cannot be inferred across the entire population, as widespread use of statins have been demonstrated to increase Lp(a) concentrations by 10–20% [26, 43]. Statin use may cause cholesterol to “escape” coordination with LDL-c receptors to form more Lp(a) [44], which indicates a need to monitor populations that are treated with statins.

The impact of Lp(a) on ARAS has been raised and been seriously questioned in previous studies, as both positive and negative results have been reported [25, 26, 45, 46]. Park et al. [45] performed renal arteriography at the time of cardiac catheterization in 270 patients and screened 28 ARAS (≥ 50% narrowing of renal artery) and 242 non-ARAS patients, concluding that Lp(a) was not associated with ARAS (median, ARAS: 143 mg/L vs. non-ARAS: 188 mg/L). In contrast, Scoble et al. [46] examined the lipoprotein profiles in a small number of patients with (*n* = 32, ≥ 30% narrowing of renal artery on angiography) or without (*n* = 32, matched with ARAS patients for clinical baseline features but no angiography performed) ARAS in a case-controlled study, revealing that serum Lp(a) levels were higher in the non-ARAS group (mean ± SD, ARAS: 310 ± 210 mg/L vs. non-ARAS: 580 ± 450 mg/L; *P* < 0.01). The negative relationship between ARAS and Lp(a) was explained by an Apo (a) polymorphism. Zhang et al. [25] performed a cross-sectional study of 1200 Chinese patients who underwent renal arteriography immediately after coronary angiography, and found that Lp(a) was significantly higher in patients with mild and advanced ARAS (≥ 30% narrowing of renal artery) by univariate logistic regression (percentage of high serum Lp(a), ARAS: 24.2% vs. Non-ARAS: 17.5%; *P* = 0.039). Catena et al. [26] examined 50 hypertensive patients with ARAS (in those with mild and advanced ARAS (≥ 70% narrowing of renal artery on angiography) and 58 hypertensive patients with comparable cardiovascular risk factor burden but non-ARAS (assessed by angio-MRI or angio-CT scan and/or renal angiography) in a cross-sectional study, which demonstrated that Lp(a) levels in the highest tertile had greater risk than the lowest tertile (OR: 3.70; *P* = 0.016). Further analyzing their results, we found that some studies had insufficient sample sizes for analysis, while one study diagnosed ARAS by non-invasive imaging methods, which could have resulted in variability in patient assignment. In addition, few studies have taken the effect of Lp(a) at low LDL-c levels into account, resulting in studies with insufficient information to establish coherent conclusions. In this study, angiography was used to assess a 50% narrowing of renal artery in order to classify patients in either the ARAS group or non-ARAS, as opposed to non-invasive imaging, and this meets the gold standard of diagnosis. In addition, the datas in this study were thoroughly and expansively collected compared to prior studies and therefore can provide higher quality evidence. It must be noted, however, that there are still some limitations to this study. Firstly, as this study utilized cross-sectional data studies, only correlations can be inferred, rather than causality, which established the findings as a reference tool for clinical practice. Secondly, a major concern of the study is the selection bias due to the strict inclusion and exclusion criteria regarding renal angiography. However, as a gold standard of ARAS, renal angiography provides a reliable prerequisite for the analysis of residual risk factor for ARAS. Using the strict criteria is an effective and valuable way to increase the homogeny of the high-risk ASCVD patients to study the residual effect of Lp(a) on ARAS. As the Lp(a) was shown to be significantly associated with the occurrence of ARAS even in high-risk ASCVD population of patients in the explorative study, we postulated that the effect of Lp(a) will be more robust in the population with lower risk. Further studies are warranted in more general population to screen potential ARAS by renal artery ultrasound examination, and validate the finding in the current study. Another concern of the study is that our sample size was relatively small. In the stratified analysis for LDL-c, 57 cases were available for low levels of LDL-c group, potentially limiting the statistical power to detect the associations and producing the extremely wide confidence intervals that we observed. However, using PASS v.13 (NCSS, LLC Kaysville, UT USA), we found that the study was able to achieve 75% power, which is acceptable. And we did find consistent effect trend and significant difference between the prevalence of ARAS and different concentrations of Lp(a) in low levels of LDL-c patients, even with the limited sample size. Future study with larger population is needed to increase the power of the study. Thirdly, patients with poor kidney function may also have proteinuria, causing the liver to produce more lipoprotein, including Lp(a) and potentially effecting serum Lp(a) levels.

Great progress has been made in understanding the role of Lp(a) in ARAS, but much remains to be explored. Patients under therapy have more clinical events of ARAS than are prevented, indicating that residual risk factors, such as Lp(a), need to be examined and taken into account. Given the potential CVD risks of Lp(a), treatment is now an urgent task. Great importance has been given to reducing LDL-c levels and we already have comprehensive lipid-lowering medications. Now it is high time to pay more attention to the control of Lp(a) level. The 2019 European Society of Cardiology guidelines recommend measuring Lp(a) concentration at least once in each adult person’s lifetime and consider 180 mg/dL of Lp(a) to be a very high inherited level that indicates danger for ACSVD (Class IIa, Grade C) [8]. It must be noted that, currently, no known medications or nutrients intake that can directly lower Lp(a) levels have been used [47].

## Conclusion

This study revealed that a subgroup of patients with renal artery stenosis may presented with low LDL-c levels. Under this situation, high Lp(a) concentration is independently and significantly associated with ARAS and thus is a residual risk factor. This finding could be useful for the prevention and early warning of ARAS in clinical practice. Further studies will investigate the mechanism by which the Lp(a) may be leveraged for new treatments.

## Supplementary information

**Additional file 1: Table S1.** Sensitivity analysis for adjusting the confounding effect of antihypertensive and statin.

## Data Availability

The data set analyzed in this study can be reasonably obtained from the corresponding author.

## Abbreviations

ASCVD: Arteriosclerotic cardiovascular disease
CVD: Cardiovascular disease
Lp(a): Lipoprotein (a)
ARAS: Atherosclerotic renal artery stenosis
PAD: Peripheral arterial disease
CAD: Coronary artery disease
BMI: Body mass index
SBP: Systolic blood pressure
DBP: Diastolic blood pressure
LDL-c: Low-density lipoprotein-cholesterol
eGFR: Evaluated glomerular filtration rate
DM: Diabetes mellitus
RALL: Renal arterial lumen loss
aOR: Adjusted odds ratio
ACEIs: Angiotensin converting enzyme inhibitors
ARBs: Angiotensin receptor blockers
