# Supplementary material for: Lipoprotein (a) as a residual risk factor for atherosclerotic renal artery stenosis in hypertensive patients: a hospital-based cross-sectional study
Source: Lipids Health Dis. 2020 Jul 23;19:173. doi: 10.1186/s12944-020-01272-0 (PMC7379345; doi:10.1186/s12944-020-01272-0)
Supplement: Supplementary file 1 — Additional file 1: Table S1. Sensitivity analysis for adjusting the confounding effect of antihypertensive and statin. [file 12944_2020_1272_MOESM1_ESM.doc]

**Additional file 1**

**Table S1** Sensitivity analysis for adjusting the confounding effect of antihypertensive and statin.

| Variable | Univariate | | Multivariate | |
| --- | --- | --- | --- | --- |
|  | OR (95%CI) | P value | OR (95%CI) | P value |
| Age | 1.03 (1.00, 1.07) | 0.064 | 1.03 (0.99, 1.06) | 0.165 |
| Male | 1.23 (0.65, 2.31) | 0.521 | 0.96 (0.46, 2.01) | 0.923 |
| Body Mass Index | 0.94 (0.86, 1.03) | 0.216 | 0.97 (0.88, 1.07) | 0.525 |
| Current smoking | 1.66 (0.86, 3.23) | 0.134 | 1.72 (0.80, 3.68) | 0.164 |
| Lp(a) tertile |  |  |  |  |
| Low | 1.0 |  | 1.0 |  |
| Intermediate | 0.54 (0.26, 1.15) | 0.109 | 0.54 (0.25, 1.20) | 0.130 |
| High | 1.07 (0.51, 2.25) | 0.851 | 1.28 (0.57, 2.91) | 0.549 |
| LDL-c tertile |  |  |  |  |
| Low | 1.0 |  | 1.0 |  |
| Intermediate | 0.75 (0.36, 1.58) | 0.455 | 0.80 (0.37, 1.74) | 0.571 |
| High | 0.63 (0.30, 1.32) | 0.226 | 0.68 (0.31, 1.52) | 0.349 |
| Diabetes mellitus | 0.91 (0.49, 1.68) | 0.754 | 0.75 (0.39, 1.44) | 0.381 |
| Antihypertensive | 1.00 (0.14, 7.27) | 1.000 | 1.53 (0.18, 12.80) | 0.695 |
| Statin | 0.65 (0.18, 2.39) | 0.517 | 0.23 (0.04, 1.28) | 0.092 |
